# Correction: Characterisation of morphological differences in well-differentiated nasal epithelial cell cultures from preterm and term infants at birth and one-year

**DOI:** 10.1371/journal.pone.0211611

**Published:** 2019-01-25

**Authors:** Helen E. Groves, Hong Guo-Parke, Lindsay Broadbent, Michael D. Shields, Ultan F. Power

There are errors in the Author Contributions. The correct contributions are: Conceptualization: MDS, UFP, HEG. Data curation: HEG, LB, HGP. Formal analysis: HEG, LB, MDS, UFP. Funding acquisition: MDS, UFP, HEG. Investigation: HEG, HGP, LB, UFP, MDS. Methodology: HGP, HEG, LB, UFP, MDS. Supervision: MDS, UFP. Writing–original draft: HEG. Writing–review & editing: HGP, LB, MDS, UFP.
