# Supplementary material for: Characterisation of morphological differences in well-differentiated nasal epithelial cell cultures from preterm and term infants at birth and one-year
Source: PLoS One. 2018 Dec 5;13(12):e0201328. doi: 10.1371/journal.pone.0201328 (PMC6281239; doi:10.1371/journal.pone.0201328)
Supplement: S1 Fig — (DOCX) [file pone.0201328.s001.docx]

**One year repeat Nasal brushing samples**

Unique Patient ID _______________________

Date of sample _______________________

Age of infant _______________________

PMH

Bronchiolitis Yes/No

Confirmed RSV Yes/No

Hosp attendances Yes/No

Details of each admission: _____________________________________________________________________________________________________________________________________________________________________________________________________________________________________________________

GP attendances Yes/No

Details of each attendance: ______________________________________________________________________________________________________________________________________________________________________________________________________________________________________________________

Wheezing/recurrent URTIs Yes/No

Hay fever Yes/No

Allergies Yes/No

Immunisations UTD Yes/No

Medications/antibiotics Yes/No ________________________________________________

Attending nursery Yes/No
